# The monomer TEC of blueberry improves NASH by augmenting tRF-47-mediated autophagy/pyroptosis signaling pathway

**DOI:** 10.1186/s12967-022-03343-5

**Published:** 2022-03-14

**Authors:** Juanjuan Zhu, Yuan Wen, Qiuling Zhang, Fei Nie, Mingliang Cheng, Xueke Zhao

**Affiliations:** 1 Department of Infection, Affiliated Hospital of Guizhou Medical University, No. 28, Guiyi Street, Guiyang, 550001 Guizhou China; 2 School of Clinical Medicine, Guizhou Medical University, Guiyang, 55000 Guizhou China; 3 Guizhou Botanical Garden, Guiyang, 550001 Guizhou China

**Keywords:** NASH, TsRNA, Blueberry, Autophagy, Pyroptosis

## Abstract

**Background:**

Nonalcoholic steatohepatitis (NASH) is one of the most common liver diseases and has no safe and effective drug for treatment. We have previously reported the function of blueberry, but the effective monomer and related molecular mechanism remain unclear.

**Methods:**

The monomer of blueberry was examined by ultra performance liquid chromatography-mass spectrometry (UPLC-MS). The NASH cell model was constructed by exposing HepG2 cells to free fatty acids. The NASH mouse model was induced by a high-fat diet for 12 weeks. NASH cell and mouse models were treated with different concentrations of blueberry monomers. The molecular mechanism was studied by Oil Red O staining, ELISA, enzyme activity, haematoxylin–eosin (H&E) staining, immunohistochemistry, immunofluorescence, western blot, RNA sequencing, and qRT-PCR.

**Results:**

We identified one of the main monomer of blueberry as tectorigenin (TEC). Cyanidin-3-O glucoside (C3G) and TEC could significantly inhibit the formation of lipid droplets in steatosis hepatocytes, and the effect of TEC on the formation of lipid droplets was significantly higher than that of C3G. TEC can promote cell proliferation and inhibit the release of inflammatory mediators in NASH cell model. Additionally, TEC administration provided a protective role against high-fat diets induced lipid damage, and suppressed lipid accumulation. In NASH mouse model, TEC can activate autophagy, inhibit pyroptosis and the release of inflammatory mediators. In NASH cell model, TEC inhibited pyroptosis by stimulating autophagy. Then, small RNA sequencing revealed that TEC up-regulated the expression of tRF-47-58ZZJQJYSWRYVMMV5BO (tRF-47). The knockdown of tRF-47 blunted the beneficial effects of TEC on NASH in vitro, including inhibition of autophagy, activation of pyroptosis and release of inflammatory factors. Similarly, suppression of tRF-47 promoted the lipid injury and lipid deposition in vivo.

**Conclusions:**

These results demonstrated that tRF-47-mediated autophagy and pyroptosis plays a vital role in the function of TEC to treat NASH, suggesting that TEC may be a promising drug for the treatment of NASH.

**Supplementary Information:**

The online version contains supplementary material available at 10.1186/s12967-022-03343-5.

## Introduction

Non-alcoholic steatohepatitis (NASH) is the most common chronic liver disease in the world, and the incidence is increasing year by year [1]. NASH is based on inflammation, liver steatosis, liver cell damage and fibrosis of varying degrees, which can progress to liver cirrhosis or even liver cancer [2]. However, there is a lack of clinically effective radical drugs at the moment. Therefore, there is an urgent need to have a deeper understanding of the etiopathogenesis of NASH, and to find effective treatment drugs to alleviate the pathogenesis.

Autophagy, a self-degradation process of cells, which degrade cellular proteins and damaged or excessive organelles through the formation of double membrane autophagosomes, plays a role in energy balance and cytoplasmic quality control [3, 4]. In recent years, increasing evidence observed that the autophagy flux is blocked in the livers of NASH patients [5, 6]. Additionally, autophagy defects in hepatic sinusoidal endothelial cells in patients with NASH promote the development of early liver inflammation, endothelial-mesenchymal transition, apoptosis and liver fibrosis in NASH [7]. Thioredoxin-interactingprotein, a key mediator of cellular stress response, directly interacted with p-PRKAA, leading to inactivation of mTOR and nuclear translocation of TFEB, thereby promoting autophagy and improving liver steatosis, inflammation and fibrosis [8]. All these evidences demonstrated that autophagy is closely related to liver disease, which is a potential new therapeutic target. However, the mechanism of how autophagy regulates NASH patients remains unclear.

Pyroptosis is a new type of inflammatory cell death, which mainly relies on the inflammasome composed of NLR family pyrin domain containing 3 (NLRP3), ASC and pro-caspase 1 [9]. In NASH, lipotoxicity, organelle stress, and liver cell death triggered inflammasome activation, while excessive activation of NLRP3 inflammasomes exacerbated liver steatosis [10]. Moreover, the activation of NLRP3 inflammasome leaded to pyroptosis of mouse and human primary hepatocytes, promoted IL-1β secretion, and induced stellate cell activation and liver fibrosis [11]. In recent years, the regulatory relationship between autophagy and pyroptosis has been reported. For example, inhibition of autophagy could induce LDH release, NLRP3 inflammasome activation and pyroptosis [12]. Liraglutide ameliorates NASH by prohibiting NLRP3 inflammasome and pyroptosis through mitophagy activation [13]. The above studies indicated that the process of autophagy inhibiting pyroptosis plays a crucial part in the progress of NASH. However, the molecular mechanism regulating the “autophagy / pyroptosis signal pathway” is still poorly understood. Therefore, studying this signal pathway will help us to understand the pathogenesis of NASH, which is of great significance for finding new treatment strategies for NASH.

tRNA-derived small RNA (tsRNA) is a novel regulatory non coding small RNA, which exert an important influence in multifarious biological processes [14]. tsRNA has been reported to be involved in the process of many diseases, including various cancers, acute cerebral infarction, bone metabolism related diseases, etc. [15]. Currently, a study has reported that tsRNA plays an important role in non-alcoholic fatty liver disease (NAFLD) [16]. Simultaneously, we also proposed that tsRNA plays a vital role in NAFLD cell models and mouse models via regulating autophagy [17]. However, the specific role and molecular mechanism of tsRNA in NASH remain to be elucidated.

In recent years, as an alternative to drug intervention, dietary strategies to improve NASH have been increasingly popular. In previous research, we manifested that blueberry significantly reduced liver cell apoptosis and fat accumulation, enhanced lipid metabolism and improved fatty liver, nevertheless, it is not yet clear which active ingredients the blueberry mainly uses to improve fatty liver [18–20]. It has been reported that cyanidin-3-O-glucoside (C3G), one of the most abundant monomers in vaccinium oxycoccus pigment, improved hepatic steatosis in mice [21, 22]. Furthermore, tectorigenin (TEC) was also one of the active ingredients of blueberries, which inhibited the expression of toll-like receptor-4 (TLR4) and the activation of the MAPK/NF-κB pathway to promote autophagy and protect liver failure [23]. These analysis emphasized the need to estimate the reasonable utility of blueberry monomer in NASH treatment and to research the mechanisms involved. Herein, we further explore which active substances in blueberries can improve the function of NASH by autophagy and pyroptosis; and focus on whether blueberry monomer relies on tsRNA to regulate the connection between autophagy and pyroptosis, in order to provide safe and effective potential drugs for NASH treatment, and provide a strong theoretical and experimental basis for the prevention and treatment of NASH.

## Materials and methods

### Extraction of active components in blueberry

First, we smashed the blueberries with a homogenizer. Then, we took 0.5 g blueberry homogenate and used 5 ml methanol solution (Fisher) containing 0.1% formic acid (Sigma-Aldrich) for ultrasonic extraction for 15 min. Next, centrifuged for 20 min at 10 °C and 12,000 r/min, and take the supernatant. Finally, the liquid phase was detected after filtration by microporous membrane (0.45 μm).

### Detection of monomers by ultra performance liquid chromatography combined-mass spectrometry (UPLC-MS)

We extracted monomers from blueberries with methanol and distilled water respectively. When extracting with methanol, blueberry and 0.1% formic acid methanol were 1:4, then grind in a mortar. Next, the homogenate was centrifuged at 3500 rpm/min for 5 min, and took the supernatant into the liquid phase for detection, and the injection volume was 1 μl. Water extraction: blueberry and distilled water was 1:4, and the remaining steps were consistent. Finally, blueberry monomers were prepared into 1000 ppb, 800, 600, 400, 200 and 100 ppb with 0.1% formic acid methanol solution. The standard curve was drawn according to the peak area and concentration by UPLC-MS.

### Cell culture and treatment

Human hepatoma cell line HepG2 was obtained from the Chinese Academy of Sciences (CAS). HepG2 cells were cultured in DMEM (Dulbecco’s modifified Eagle’s medium) added with 10% fetal bovine serum (Biological Industries, 1707254) and antibiotics (Hyclone, HYC-SV30010) under a cell incubator with 5% CO_2_ at 37 °C.

In order to model NASH cells, HepG2 cells were cultured with 0.5 mM concentration of FFA [24]. In experimental group, NASH cell model were treated with 25, 50 and 75 μM TEC, respectively, and the selection of TEC concentration was based on others report [25]. To block autophagy, hepatocytes were cultured with 5 mM 3-Methyladenine (3MA) [26]. To knockdown tRF-47-58ZZJQJYSWRYVMMV5BO (tRF-47) in vitro, tRF-47 antagomir was transfected into TEC-treated steatosis hepatocytes using Lipofectamine 2000 (Invitrogen, USA).

### Cell viability assay

The Cell Counting Kit-8 (CCK8, Dojindo, CK04) was utilized to assess the viability of HepG2 cells. Cells were cultured with FFA in 96-well plates (1 × 10^4^ cells/well) and treated with different doses of TEC for 24 h. Then, we added CCK8 reagent, and put the plate into 37 °C incubator for 4 h. The absorbances in different groups were detected at 450 nm. In the blank group, the well only contained medium, and the cells without any treatment were used as the control group.

### Western blot analysis

We lysed cells with RIPA buffer (beyotime, China) and extracted proteins from cells. Then, BCA assay kit (Invitrogen) measured the protein concentration. 10% SDS-PAGE was employed to separate proteins. The protein was transferred to the PVDF membrane (Bio-Rad, USA), the PVDF membrane was blocked with 5% milk, and then incubated with a primary antibody. And then incubated with an NLRP3 (Abcam; ab263899; dilution 1:1000); GSDME (Abcam; ab215191; dilution 1:1000); or GAPDH antibody (Abcam; ab181602; dilution 1:10,000) at 4 °C overnight. After washing the membrane, it was incubated with goat anti-rabbit IgG-HRP for 1–2 h. The photos were taken using Odyssey infrared Fluorescence Western blotting system (Li-Cor Biosciences), and the quantitative pictures was obtained via ImageJ software.

### LDH release assay

According to the manufacturer’s protocol, LDH levels in the cell supernatants were determined using an LDH Cytotoxicity Assay Kit (Beyotime, C0016). The absorbance was read at 490 nm with a microplate reader (Thermo Fisher Scientific).

### RNA isolation, small RNA libraries construction, and sequencing

The total RNA was obtained from the FFA group and the FFA + TEC group of HepG2 cells (n = 3 for each group) by the RNeasy Mini Kit (Qiagen, Hilden, Germany). The quality of RNA was detected by the 1% agarose gel electrophoresis. The concentration and integrity of total RNA were measured by the NanoDrop ND-1000 spectrophotometer (Thermo Fisher Scientific, Waltham, USA) and Agilent 2100 Bioanalyzer (Agilent Technologies, Santa Clara, USA). Following passing the quality control tests, the Multiplex Small RNA Library Prep Set for Illumina (NEB, MA, USA) was leveraged to establish the library following the manufacturer’s protocol. Briefly, the rRNA was removed and the remaining RNA was cut into small pieces. RNase H-reverse transcriptase (NEB, MA, USA) was used to synthesize the first-strand cDNA and the end repair was conducted, then the fragments were sorted and PCR was performed to amplify the cDNA. An Illumina Hiseq2500 platform (Illumina, San Diego, California) was used for the sequencing of library preparations.

### Bioinformatic analysis

Filtered out short (< 15 nt) and low-quality reads from the original sequence. Then, clean reads were performed through miRBase database (https://mirbase.org/), piwi-interacting RNA database, NCBI (https://www.ncbi.nlm.nih.gov/), Genomic tRNA database (http://gtrnadb.ucsc.edu/) and tRFdb (http://genome.bioch.virginia.edu/trfdb/) and Mintbase to identify known miRNAs and tRFs. RNAhybrid (https://bibiserv.cebitec.uni-bielefeld.de/rnahybrid/submission.html/) and miRanda (http://www.microrna.org/) were used to predict targets of the differentially expressed tsRNA. tsRNA were defined as differentially expressed tsRNAs when |fold change|> 1 and FDR < 0.05 using the DE-Seq2.0 algorithm. Gene Ontology (GO) and Kyoto encyclopedia of genes and genomes (KEGG) pathway analysis were used to analyze functions of the differentially expressed tsRNAs targets. A tsRNA/ mRNA/pathway interaction network was constructed using Cytoscape 2.8.3.

### Quantitative real-time polymerase chain reaction (qRT-PCR)

The total RNA were extracted using Trizol reagent (Invitrogen, CA, USA) and subjected to obtain cDNA by PrimeScript™ RT reagent kit (Takara, China). qRT-PCR analysis was performed using Fast SYBR Green master mix (Life Technologies, USA). U6 and GAPDH served as an internal control. The qRT-PCR conditions used were: 1 cycle at 95 °C for 10 min, followed by 40 cycles at 95 °C for 5 s, and annealing and extension at 57 °C for 30 s. The relative expression were calculated using the 2^−∆∆Ct^ method. The primer sequences were presented in Additional file 1: Table S1.

### Animals and treatment

The male C57BL/6 mice (8–10 week old, 20–25 g) were purchased from the Animal Center of Guiyang Medical College, and randomly divided into 5 groups (n = 5). The animal model was constructed based on the method recently described by others [23, 27]. The first group was the control group: mice were fed with standard feed. The second group was the NASH group: mice were fed with the compound high fat feed (88.3% common feed + 10% lard + 1.5% cholesterol + 0.2% sodium cholate). The remaining three groups were low, medium and high drug groups: the mice were administered with TEC by gavage at 7.5, 15.0 or 30.0 mg/kg once per day for 6 weeks after 10 weeks of high fat feeding [27, 28]. To knockdown tRF-47 in vivo, tRF-47 antagomir (5 μg/mouse in 1.5 mL saline) was injected into the tail vein of NASH mice 3 times per week for 2 weeks after 9 weeks of high fat feeding [17]. At the end of the experimental period, the animals were euthanized, and the livers and serum were immediately processed for pathological examinations and biochemical analyses.

### Biochemical analysis

The serum samples were used for biochemical analysis. The levels of aspartate aminotransferase (AST) and alanine aminotransferase (ALT) were determined by a Biochemical Analyzer (Siemens Advia 1650, Germany). The level of malondialdehyde (MDA) was detected by the MDA kit (Nanjing Jiancheng, China).

### Haematoxylin–eosin (H&E) staining

Liver tissues were fixed in 10% neutral buffered formalin, dehydrated in graded volumes of ethanol alcohols, embedded in paraffin, sectioned at 3 μm slices, and stained with hematoxylin and eosin (H&E) for microscopic examination.

### Triglyceride content detection

Triglyceride (TG) detection kit (Nanjing Jiancheng, China) was used to analyze the level of liver TG. Briefly, a small portion of liver tissue (50 mg) was collected and homogenized in 100% ethanol (450 ml). After centrifugation, glycerol lipase oxidase method was used for analysis. Samples were reacted with the mixture from the kit and were incubated at 37 °C for 10 min, and absorbance at 510 nm was read with a microplate reader (Thermo Fisher Scientific).

### Total cholesterol content detection

Total cholesterol (TC) levels in liver were analyzed using a CheKine™ Total Cholesterol Colorimetric Assay Kit (Abbkine, KTB2220). Liver tissues were harvested and lysed with the buffer. Subsequent procedure was performed with the kit according to protocol and the samples were read with spectrometer at 500 nm. Before measurement, we first standardize the cholesterol concentration and draw the standard curve.

### Immunohistochemistry

Immunohistochemistry for NLRP3 and gasdermin E (GSDME) was conducted, the tissues sections were blocked with 8% goat serum in PBS, and then incubated with anti-NLRP3 antibody (Abcam, ab214185, dilution 1:100) and anti-GSDME antibody (Abcam, ab230482, dilution 1:500) at 4 ˚C for 12 h. Thereafter, the sections were incubated with anti-rabbit IgG H&L (HRP) (Abcam, ab6721, dilution 1:1000) at room temperature for 1 h. Finally, the sections were examined under a light microscope and Nikon Photo-Imaging system (H550L, Tokyo, Japan).

### Oil Red O staining analysis

The Oil Red O Kit was utilized to stain TG. 5 μm thick frozen sections of liver tissue or HepG2 cells were washed twice with PBS and fixed in 4% paraformaldehyde at room temperature for 40 min. After two washes in 60% isopropyl alcohol, the slices or cells were stained with an Oil Red O stain kit (KeyGEN, KGA329) according to the manufacturer’s protocol. After removing the Oil Red O solution, the slices or cells were washed with distilled water five times and observed under a microscope.

### Enzyme-linked immunosorbent assays

Following the instruction of the ELISA kit (Mlbio), we used the enzyme-linked immunosorbent assays to quantify IL-6, TNF-α, IL-10, IL-4, IL-17, and IL-1β levels in liver tissue or HepG2 cell supernatants.

### Analysis of immunofluorescently-stained

The liver tissue or HepG2 cells were fixed with 4% paraformaldehyde. The samples were blocked with 5% BSA in PBS at room temperature (RT) for 1 h, and then incubated with rabbit monoclonal anti-LC3B IgG (Abcam, EPR18709) at 1 µg/ml overnight. This was followed by incubation with fluorescein isothiocyanate-conjugated goat anti-rabbit IgG (Abcam, ab150077) at RT for 1 h. 4’, 6-Diamidino-2-phenylindole (DAPI) was used to stain the nuclei. We used a microscope (Olympus Corporation) to view the samples.

### Statistical analysis

The data were expressed as the mean ± standard deviation (SD). All the groups were compared using analysis of one-way analysis of variance (ANOVA) or *t*-test. SPSS 17.0 (SPSS, USA) statistical software and Graphpad Prism 8.0. (La Jolla, USA) were used to analyze these data, and *p* < 0.05 was regarded as statistically significant.

## Result

### TEC is the main blueberry monomer to improve NASH in vitro

In previous research, we have found that blueberry can significantly improve the liver pathology of NASH mouse, but it is not clear which component of blueberry plays a role [18]. In order to explore the effective active components of blueberry, we previously determined the components by pH differential method and found that procyanidins were the most abundant in blueberry [18]. We then used FFA (0.5 mM) to treat HepG2 cells as a useful in vitro NASH model. Anthocyanins were further extracted from blueberries and treated with different concentrations of anthocyanins for hepatic steatosis HepG2. Oil red O staining showed that anthocyanin could inhibit the formation of lipid droplets (Additional file 2: Fig. S1A).

**Fig. 1:**
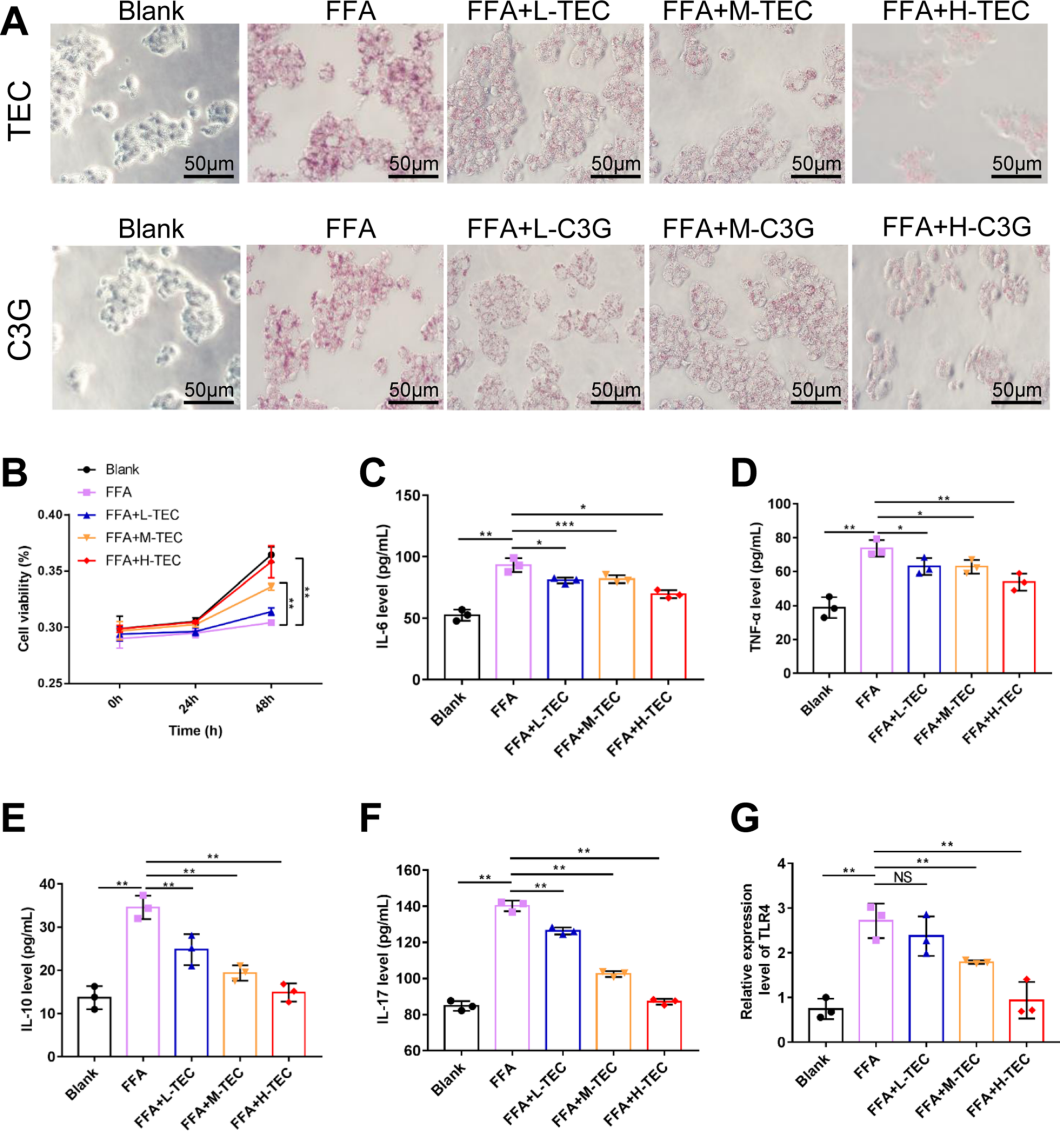
TEC alleviated lipid production and inflammatory response of steatotic hepatocytes. **A** Lipid droplets were stained by Oil Red O staining. Scale bar = 50 μm. **B** Cells were cultured for 0, 24 and 48 h, then were analyzed with CCK-8. **C**–**F** The expression of inflammatory factors was detected by ELISA. **G** The expression level of TLR4 using qRT-PCR. The values are expressed as mean ± SD.*P<0.05, **P<0.01, ***P<0.001, *NS* No significance (n=3)

In order to study which monomer anthocyanins play a role, we have determined the five most abundant monomers in blueberry anthocyanins by UPLC (Additional file 2: Fig. S1B), including C3G, myricetin, myricetin 3-o-galactoside, delphinidin and TEC (Additional file 1: Table S2). Then we used different concentrations of these five monomers to separately treat the NASH model, and found that only C3G and TEC can inhibit the formation of lipid droplets, but TEC was more effective than C3G (Fig. 1A). The CCK-8 assay demonstrated that both high and medium concentrations of TEC can promote cell viability in the NASH model as time changes (Fig. 1B). Then we used ELISA to detect the expression of inflammatory factors, which showed that different concentrations of TEC could reduce the release of IL-6, TNF-α, IL-10, IL-17, and IL-4 (Fig. 1C–F, Additional file 3: Fig. S2A). In addition, studies have shown that IL-17 induces disease-related tissue inflammation through TLR4 signaling pathway activation [29]. Therefore, we detected the expression of TLR4 and found that compared with the control group, TLR4 expression increased in NASH model, while TEC eliminated this upward trend (Fig. 1G). These results suggested that TEC can improve the pathological characteristics and inflammatory response of NASH in vitro.

### TEC improves fatty liver in vivo

**Fig. 2:**
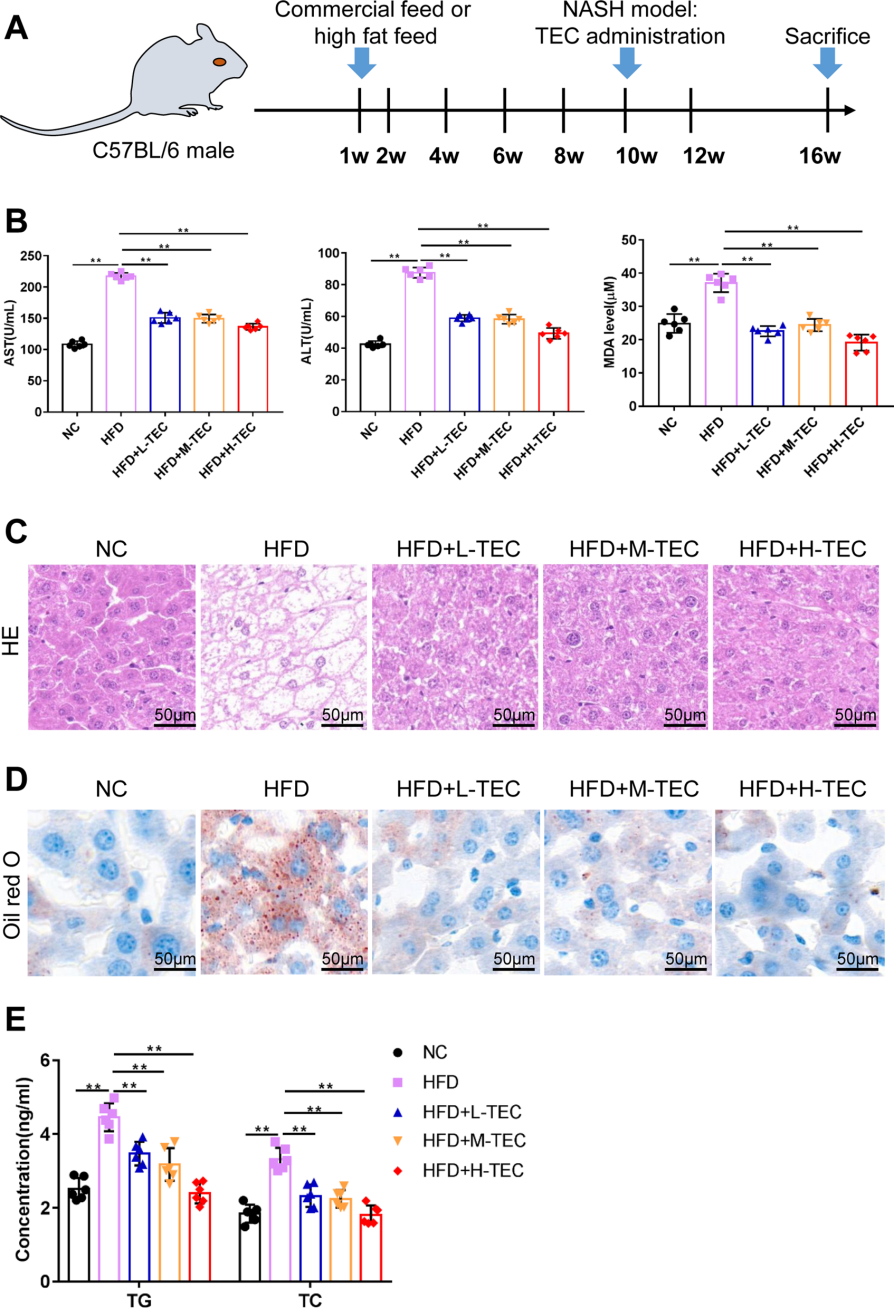
TEC improves fatty liver in vivo. **A** Schematic diagram of animal model. **B** Effects of TEC on the activity AST, ALT and MDA in serum. The representative micrographs of H&E staining **C** and Oil Red O staining (**D**). Scale bar = 50 μm. **E** The content of TG and TC. **P<0.01 (n=6)

We constructed NASH mice with high-fat diet (HFD) and intervened with different concentrations of TEC to further explore the therapeutic effect of TEC on NASH in animals (Fig. 2A). Tissue examination showed that TEC significantly protected the liver of NASH mice from damage, including reducing the levels of ALT, AST and MDA (Fig. 2B). The H&E (Fig. 2C) and Oil-red O (Fig. 2D) staining showed that compared with the control group, the liver tissue of the HFD group was significantly damaged, and there were obvious fat vacuoles and increasing lipid droplets, while with the increase of TEC addition, the TEC treatment markedly alleviated liver steatosis in high-fat diet mice. Similarly, the results of TG and TC also proved that TEC could significantly reduce lipid accumulation (Fig. 2E). The above results showed that TEC had a specific therapeutic effect on fatty liver in NASH mice.

### TEC regulates autophagy and pyroptosis in vivo

**Fig. 3:**
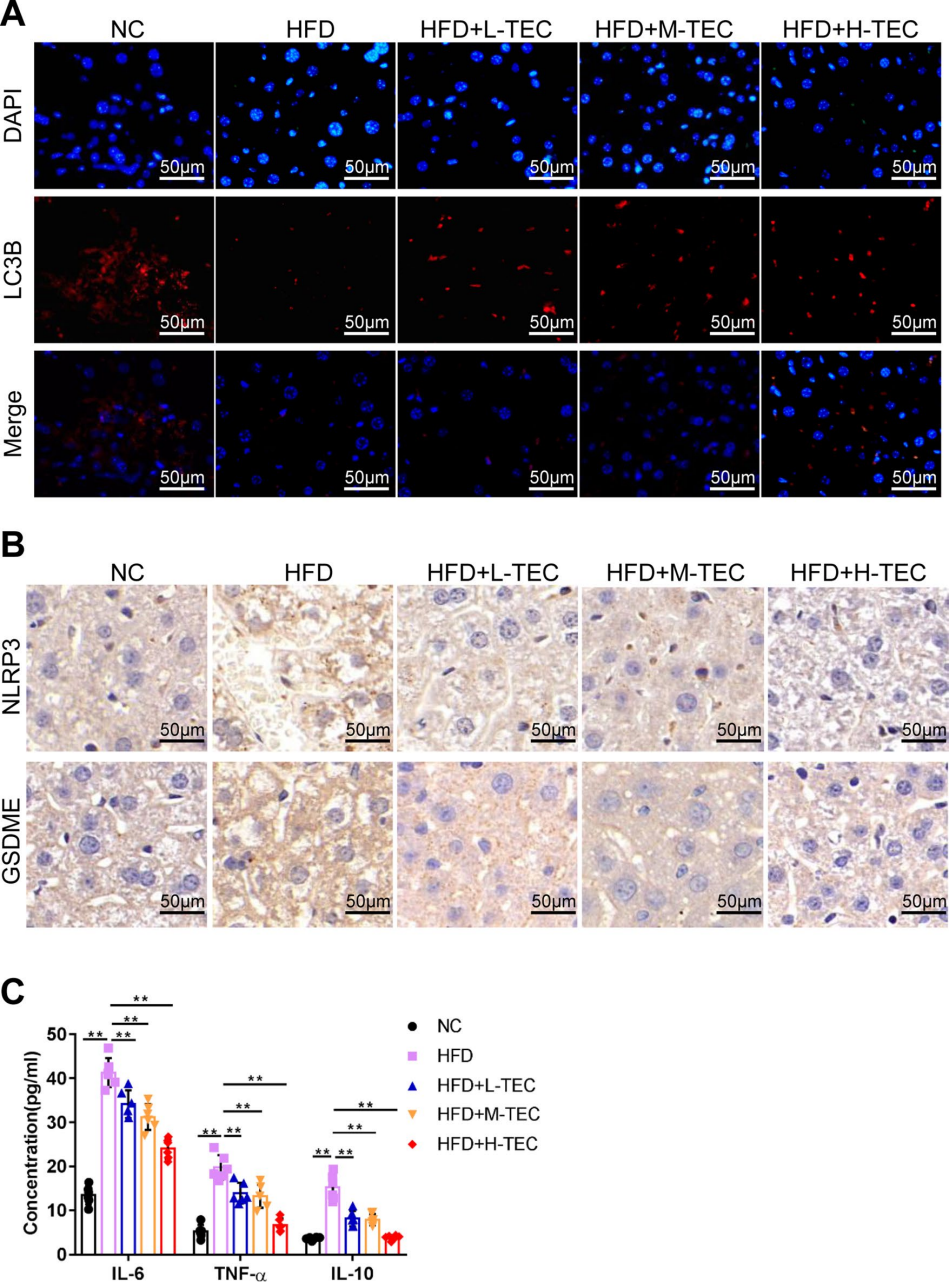
TEC promoted autophagy and inhibited pyroptosis in NASH mice model. **A** Fluorescence microscope to detect the LC3B expression. Scale bar = 50 μm. **B** The validation of the protein expression of NLRP4 and GSDME using immunohistochemical staining. Scale bar = 50 μm. **C** The expression of IL-6, TNF-α and IL-10 was measured by ELISA detection. **P<0.01 (n=6)

We have previously reported that autophagy is reduced in NAFLD mice, and autophagy agonists can improve NAFLD, which prompted us to explore whether TEC affects autophagy [17]. In vivo, TEC significantly enhanced the level of autophagy marker LC3B in the liver of NASH mice (Fig. 3A). In addition, because pyroptosis plays an important role in NASH, and TEC is closely related to inflammation, we further detected the effect of TEC on pyroptosis [12, 18]. The results showed that NLRP3 and GSDME (marker of pyroptosis) were up-regulated and the inflammasomes and TLR4 were activated in NASH mice, while TEC could significantly inhibit pyroptosis and reduce the activation of inflammasomes and TLR4 (Fig. 3B–C, Additional file 3: Fig. S2B–D). These results indicated TEC may improve NASH by activating autophagy and inhibiting pyroptosis.

### TEC inhibites hepatocyte pyroptosis depends on activation of the autophagy

**Fig. 4:**
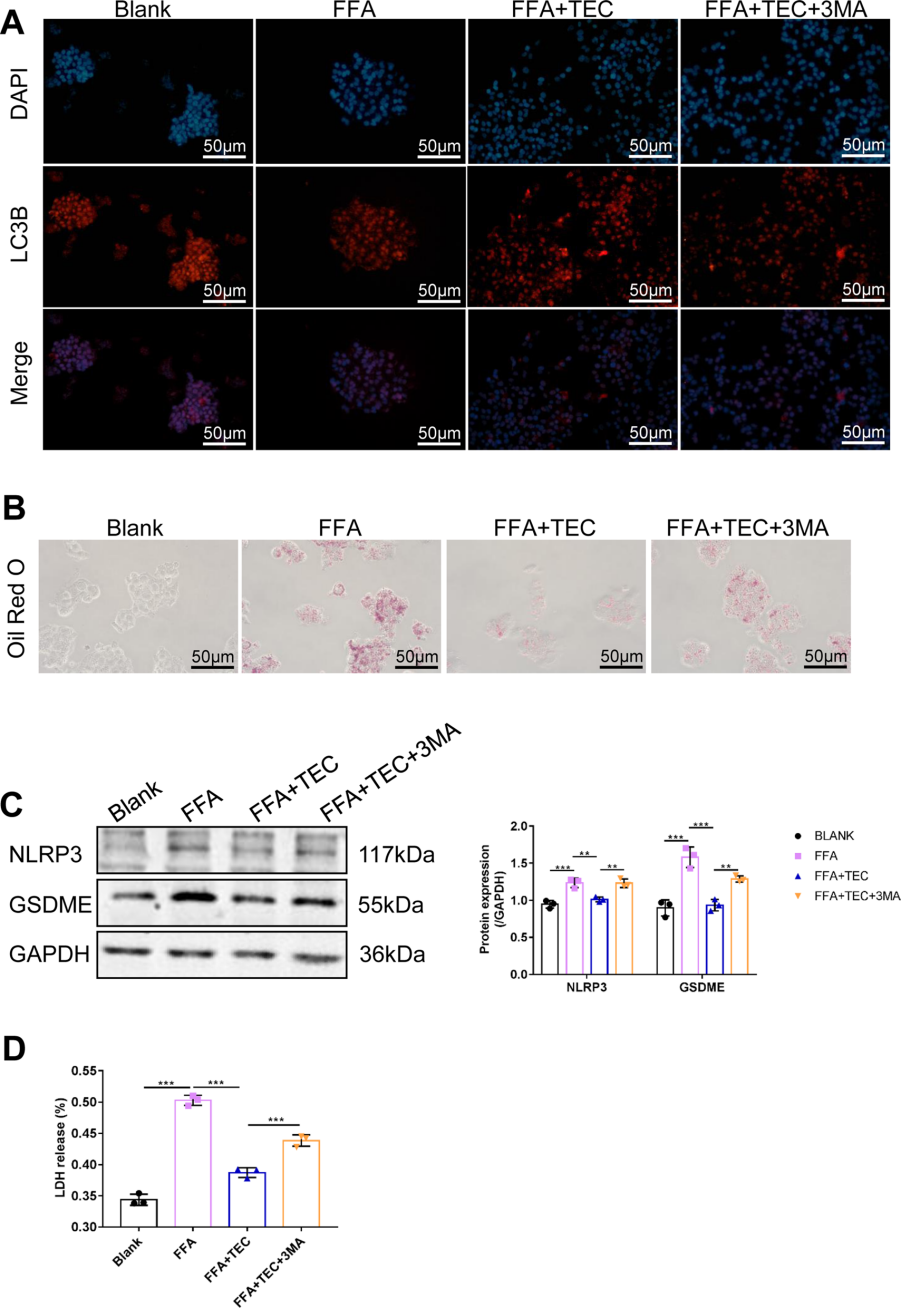
TEC inhibited pyroptosis by promoting autophagy in NASH cells. **A** The expression of LC3B in hepatocytes was detected by immunofluorescence. Scale bar = 50 μm. **B** The Oil red O staining demonstrated that TEC reduced lipid deposition through autophagy activation. Scale bar = 50 μm. **C** The NLRP4 and GSDME protein expression were detected by western blot assay. **D** The release of LDH. **P<0.01, ***P<0.001 (n=3). 3MA: autophagy inhibitor

Considering that autophagy negatively regulates pyroptosis, we inhibited the expression of LC3B using 3MA to assess the role of the autophagy in TEC-induced pyroptosis in hepatocytes [30]. As Fig. 4A shown, the levels of LC3 were increased by TEC treatment, while 3MA diminished LC3 expression (Fig. 4A). The Oil red O results demonstrated that 3MA rescued TEC-mediated inhibitory effects on lipid deposition (Fig. 4B). Additionally, compared with the FFA group, the expression of NLRP3 and GSDEM were down-regulated by TEC, while 3-MA reversed the results (Fig. 4C). Similarly, TEC inhibited the release of LDH caused by FFA, whereas 3MA restored the release of LDH (Fig. 4D). These results indicated that TEC inhibited pyroptosis by activating autophagy.

### Screening for tsRNAs associated with NASH via TEC treatment

**Fig. 5:**
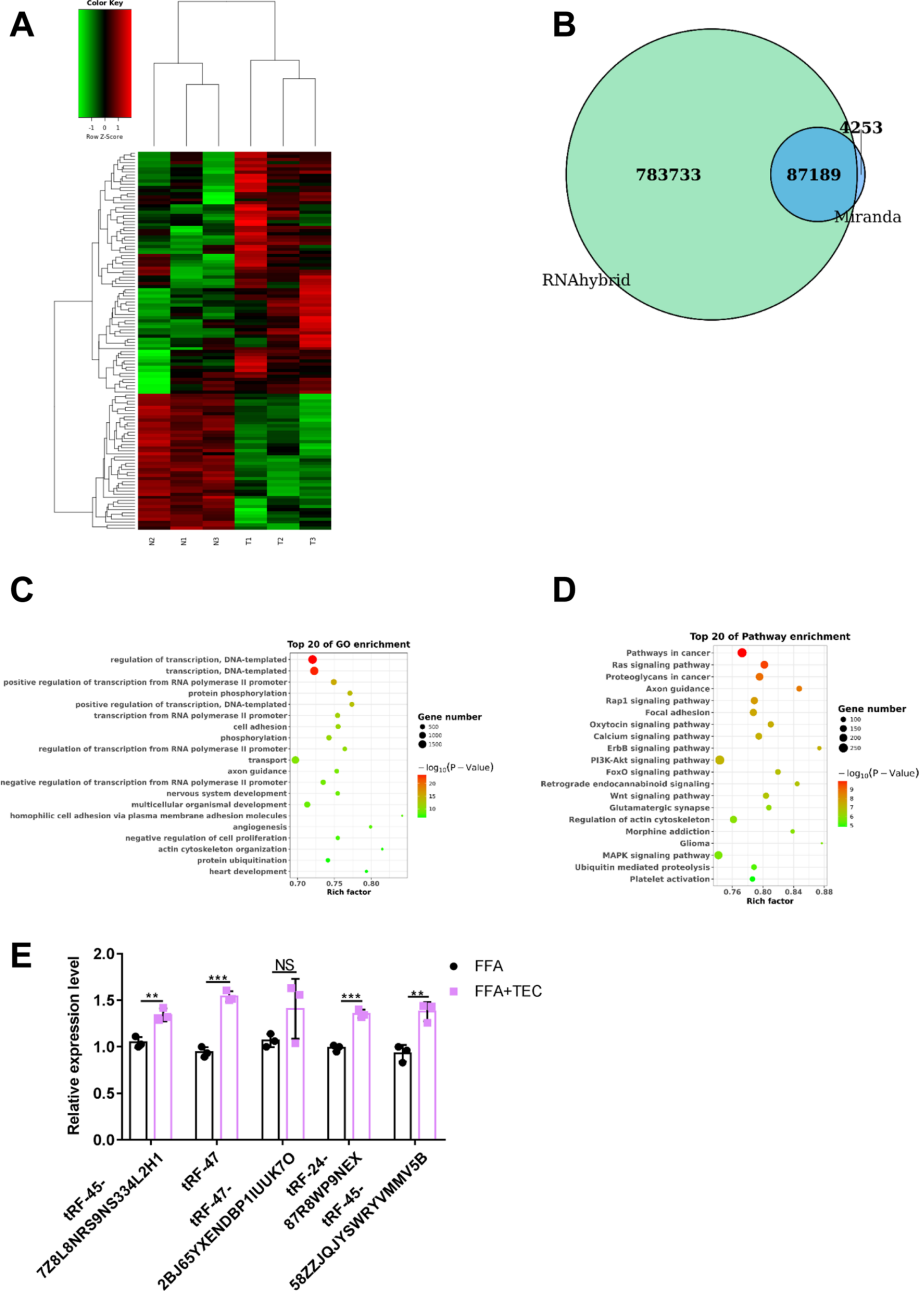
Small RNA sequencing screened tsRNA involved in TEC regulation. **A** The heat map shows the differentially expressed tsRNA between different groups (N1-3: NASH cells, T1-3: NASH cells of TEC treatment). Green: down-regulated. Red: up-regulated. **B** Venn diagrams of the number of target genes for DE-tsRNAs in two group using RNAhybrid and Miranda algorithms. **C** GO enrichment analysis of DE-tsRNAs. **D** KEGG-enriched terms of DE-tsRNAs. **E** The expression level of candidate DE-tsRNAs using qRT-PCR. The values are expressed as mean ± SD. **P<0.01, ***P<0.001, NS: No significance (n=3)

To explore abnormally expressed tsRNAs between FFA and FFA + TEC in hepatocyte, small RNA sequencing was performed. We analyzed the distinct patterns of expression of tsRNA by drawing a heat map, and found that there were 78 up-regulated tsRNA and 44 down-regulated tsRNA (Fig. 5A), among them, we listed the top ten up-regulated and down-regulated tsRNAs (Additional file 1: Table S3). To further recognize the possible functions and mechanisms of the differential expression tsRNAs (DE-tsRNAs) with TEC treated, the function analysis was performed. A total of 87,189 target genes for the DE-tsRNAs were obtained through the intersection of the RNAHybrid and miRanda algorithms (Fig. 5B). GO analysis showed some critical biological processes for DE-tsRNAs enrichment, such as “regulation of transcription, DNA-templated”, “axon guidance”, “positive regulation of transcription from RNA polymerase II promoter” and “multicellular organismal development” (Fig. 5C). Furthermore, the target genes of tsRNA were mainly associated with “PI3K-Akt signaling pathway”, “Wnt signaling pathway”, “MAPK signaling pathway” and “FoxO signaling pathway” (Fig. 5D). Finally, we chose to screen tsRNAs with large differences, stable expression, and involved in the autophagy signaling pathway for PCR verification. The results revealed that tRF-45-7Z8L8NRS9NS334L2H1, tRF-47-58ZZJQJYSWRYVMMV5BO, tRF-24-87R8WP9NEX and tRF-45-58ZZJQJYSWRYVMMV5B were up-regulated, similar to the expression trend in the tsRNAs transcriptome (Fig. 5E). Furthermore, we constructed the regulatory network of tsRNA-mRNA-pathway (Additional file 4: Fig. S3). tRF-47-58ZZJQJYSWRYVMMV5BO, which we termed tRF-47, was the highest expression level and the most obvious difference, thus we chose it for follow-up experiments.

### Attenuation of tRF-47 expression eliminates the TEC-mediated improvement effects on NASH cells

**Fig. 6:**
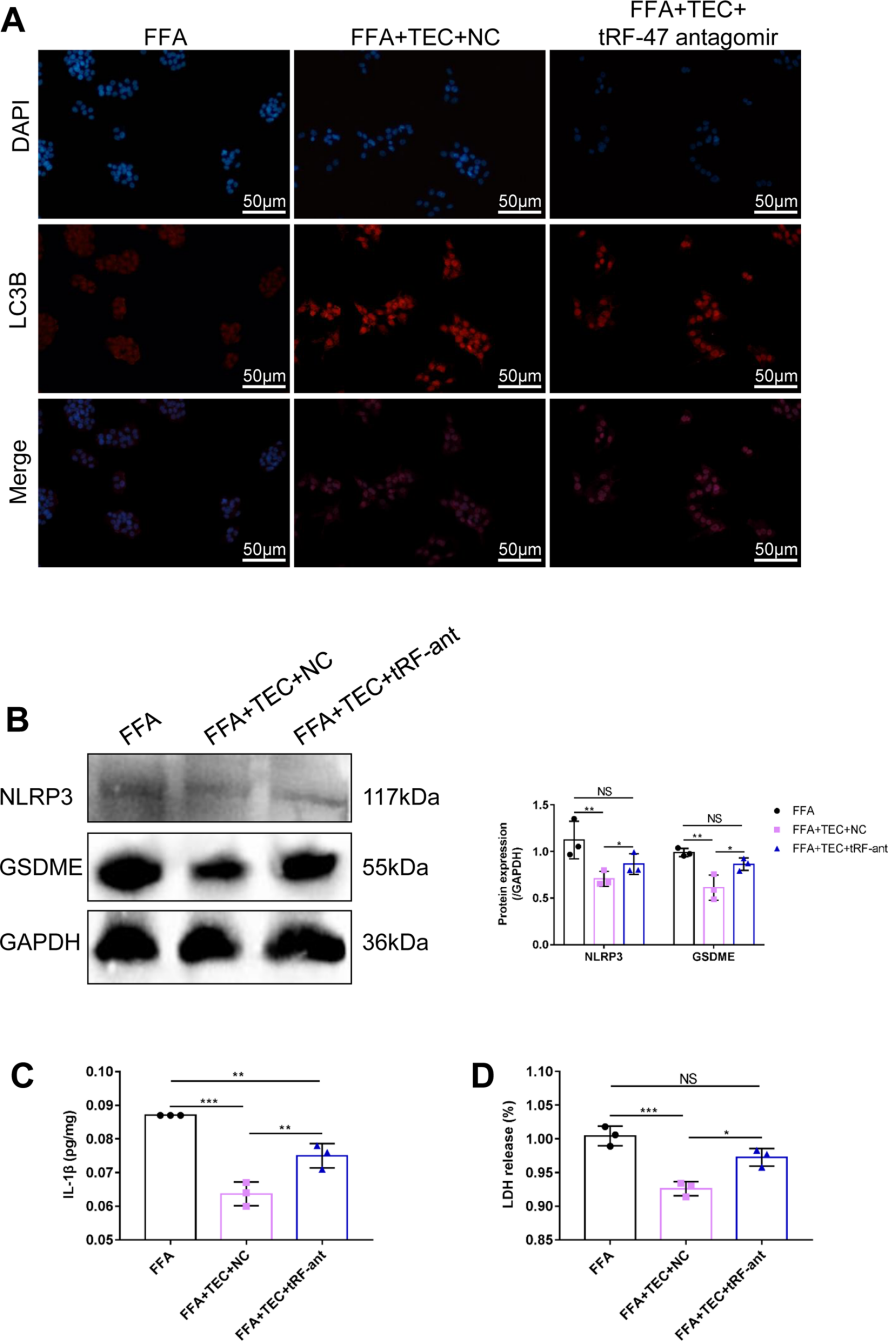
TEC enhanced autophagy and prohibited pyroptosis by up-regulated tRF-47. **A** The expression of LC3B in hepatocytes was detected by immunofluorescence. Scale bar = 50 μm. **B** The NLRP4 and GSDME protein expression detected by western blot assay. **C** The expression of IL-1β was measured by ELISA detection. **D** The release of LDH. *P<0.05, **P<0.01, ***P<0.001, NS: No significance (n=3)

To determine whether the dysregulation of tRF-47 is involved in the regulation of enhancing autophagy and inhibiting pyroptosis by TEC, we used specific tRF-47 antagomir to knock down tRF-47 expression. Compared with the control group, the reduction of tRF-47 expression partially lessened the effect of TEC in promoting the autophagy of NASH cells (Fig. 6A). Similarly, the TEC-induced reduction of NRLP3 protein was restored by tRF-47 antagomir (Fig. 6B). Besides, compared with control, TEC reduced the levels of inflammatory mediators and TLR4, and release of LDH, whereas less tRF-47 reversed this phenomenon (Fig. 6C, D, Additional file 5: Fig. S4A–C). These data manifested that TEC relies on tRF-47 to promote autophagy and weaken pyroptosis.

### TEC ameliorates NASH in vivo by tRF-47

**Fig. 7:**
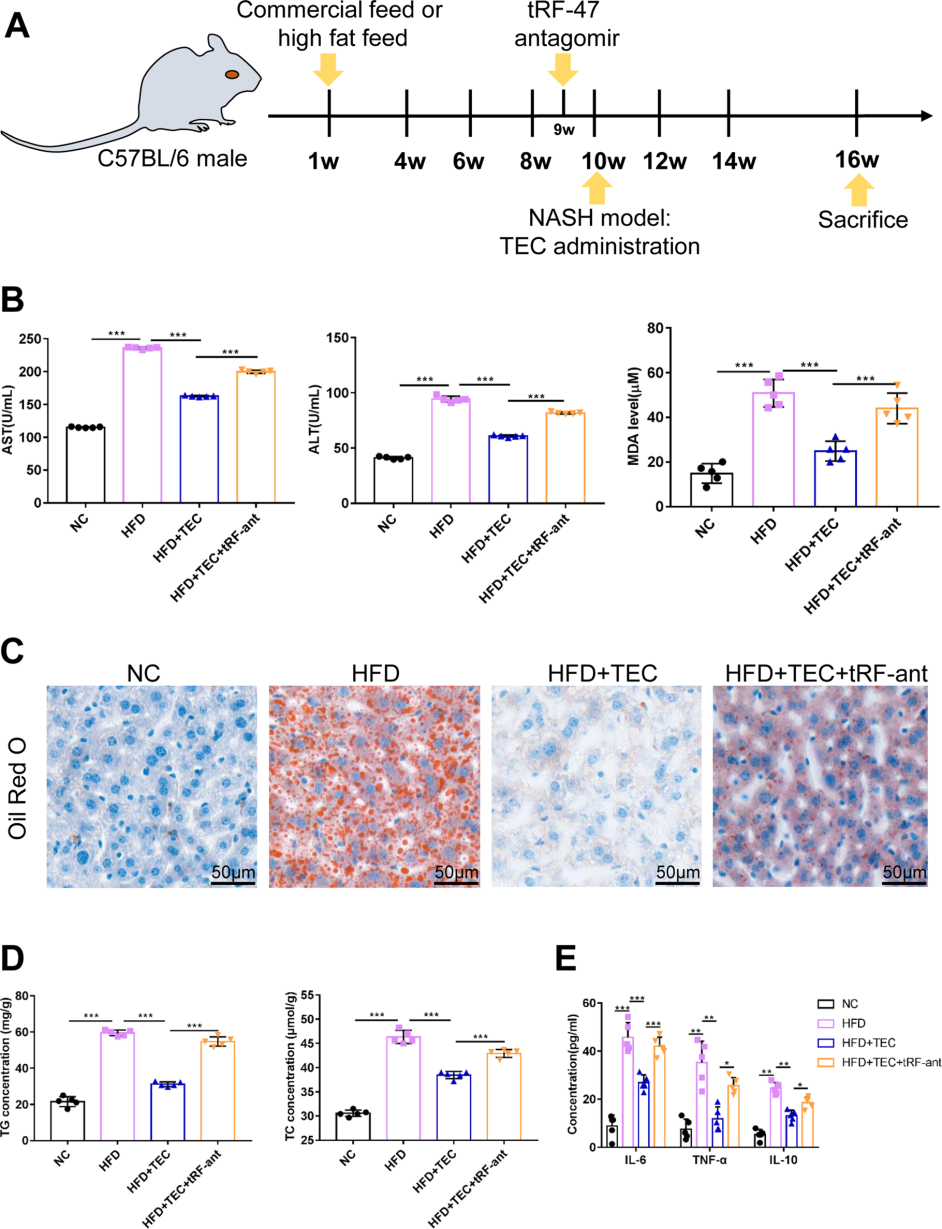
TEC ameliorated NASH *in vivo *by tRF-47. **A** Schematic diagram of animal model. **B** The activity AST, ALT and MDA in serum. **C** The representative micrographs of Oil Red O staining. Scale bar = 50 μm. **D** The content of TG and TC. **E** The expression of IL-6, TNF-α and IL-10 was measured by ELISA detection. *P<0.05, **P<0.01, ***P<0.001, NS: No significance (n=5)

To investigate the role of tRF-47 in NASH in vivo, hepatocyte were transfected with tRF-47 antagomir, and then injected subcutaneously into NASH mice (Fig. 7A). The results showed that TEC provided a protective role against high-fat diet-induced lipid damage, while tRF-47 antagomir increased the liver damage, including up-regulation of AST, ALT and MDA (Fig. 7B). Meantime, TEC reduced lipogenesis and inflammatory cytokine release, while attenuation tRF-47 expression reversed this results (Fig. 7C–E). In brief, TEC can treat NASH patients mainly through tRF-47 molecules to advance autophagy and suppress pyroptosis.

## Discussion

NASH is an emerging risk factor for type 2 diabetes and end-stage renal disease [31]. It has become the second leading cause of liver disease among adults waiting for liver transplantation in the United States, which is expected to become the most common cause of liver transplantation in the next ten years [32]. However, there are still many doubts about the cause of NASH, and there is a lack of clinically effective drugs. Therefore, it is urgent to study the pathogenesis of NASH from different angles and find new drugs to lay the foundation for the clinical treatment of NASH. In this study, TEC, one of blueberry monomers, effectively ameliorated the symptoms of NASH by enhancing the expression of tRF-47, including reducing lipid accumulation, activating autophagy, and inhibiting pyroptosis. Our research clarified the pathogenesis of NASH from a new perspective and provided a reliable scientific basis for targeted therapy of NASH.

Currently, nano based therapy has been widely used to treat a variety of diseases [33], and dietary recommendations and physical exercise remain the mainstay of NASH therapy. Happily, compounds extracted from natural products are increasingly being used in NASH because of their efficacy and low side effects [34]. Many evidence proved that vaccinium oxycoccus pigment could be used in the treatment of obesity and NASH, such as G3G, whereas other monomers of vaccinium oxycoccus pigment also have similar effects [35]. TEC has reported to inhibit adipogenesis and related genes transcription [25]. Unfortunately, the regulatory effect of TEC on NASH has not been reported. In our study, both C3G and TEC could significantly inhibit the formation of lipid droplets in steatotic HepG2 cells, but the effect of TEC on the formation of lipid droplets was significantly higher than that of C3G, which proved that TEC is a potential therapeutic agent for the treatment of NASH.

NASH is mainly characterized by excessive fat deposition in the liver and dysregulation of lipid metabolism and reactive oxygen [36, 37]. Many studies have proved that autophagy and hepatic lipid metabolism were interrelated [5]. Singh et al. [38] reported that autophagy mediates lipid metabolism by reducing the production of triglycerides and effectively prevents the development of hepatic steatosis. In addition, autophagy deficiency could contribute to oxidative stress, cause mitochondrial dysfunction, and accelerate the activation of NLRP3 inflammatory, which resulting in pyroptosis [39, 40]. TEC effectively inhibited palmitate-induced reactive oxygen species production and mitochondrial membrane potential collapse [41]. In this study, lipid accumulation and increased abundance of LC3B by activated pyroptosis (over-expressed NLRP3 and GSDME) were observed in vitro NASH model, which were consistent with the previously reported work related to impaired hepatic autophagy is associated with NASH [6]. Furthermore, in addition to alleviating the symptoms of lipid deposition in NASH models, TEC also were observed activating autophagy (increased LC3B expression), inhibiting pyroptosis and reducing the expression of inflammatory factor (IL-6, TNF-α, IL-10, IL-4, IL-17, and IL-1β) and TLR4, which is similar to other studies [23, 42, 43]. Considering the relationship between autophagy and pyroptosis, we speculated that TEC may treat NASH via regulating autophagy and pyroptosis. Consistently, autophagy inhibitors could counteract the inhibitory effect of TEC on lipid droplet formation and enhance the expression of pyroptosis protein and release of LDH. Combined with TEC improving liver failure by regulating autophagy pathway, and TEC regulated TLR4, NF-κB and MAPK pathway to inhibit the occurrence of inflammatory response, which all proved our view that TEC may improve NASH by regulating hepatocyte autophagy and pyroptosis [23, 44].

tsRNA is a non-coding small RNA which involved in a variety of physiological and pathological processes. It has been identified as a new class of NAFLD biomarkers due to the significant increase of tsRNAs in plasma of NAFLD patients [45]. Interestingly, we have previously reported that tsRNAs regulated autophagy, which played an important role in NASH [17]. In the present study, we sequenced the small RNA of hepatocytes with TEC and FFA treated, and found 122 differentially expressed tsRNAs, including 78 up-regulated and 44 down-regulated tsRNAs. These differential tsRNAs were mainly involved in “PI3K-Akt signaling pathway”, “Wnt signaling pathway”, “MAPK signaling pathway” and “FoxO signaling pathway”, which have been reported to be participated in autophagy and pyroptosis [46]. We then screened the tsRNAs involved in autophagy and pyroptosis signaling pathway for PCR verification, which showed that the expression difference of tRF-47 was the most significant after TEC treatment. Not surprisingly, tRF-47 antagomir significantly reversed the regulatory effects of TEC on autophagy and pyroptosis in vitro. Similarly, in vivo experiments have manifested that TEC improved NASH by activating tRF-47 molecule. These results demonstrated that TEC enhanced autophagy and weaken pyroptosis by activating the expression of tRF-47, ultimately reducing lipid deposition and improving NASH.

## Conclusions

This study showed that TEC had an inhibitory effect on NASH cells and animal models. We demonstrated TEC can reduce the injuries and inflammation of liver tissues and hepatocytes in NASH models by strengthening autophagy and inhibiting pyroptosis. Meanwhile, we demonstrated for the first time that TEC ameliorates NASH by targeting tRF-47 to regulate autophagy and pyroptosis. We will continue to verify it in clinical samples in the future, providing a new idea for further understanding the effect and mechanism of TEC on NASH, and a promising drug for the clinical treatment of NASH.

## Supplementary Information

**Additional file 1: Table S1** The primers and sequence. **Table S2** Determination of monomer components in anthocyanins by UPLC. **Table S3** The top ten up and down regulated tsRNAs.

**Additional file 2: Fig. S1** Analysis of active components in blueberry. **A** The effects of different concentrations of anthocyanins on lipid droplet formation in HepG2 cells were detected by Oil Red O staining. **a** Blank group: cells were not treated. **b** NASH model group: steatosis HepG2 cells with FFA (0.5 mM) induced. **c** Low concentration anthocyanin group: 0.1 mg/ml blueberry anthocyanin treated model cells. **d** Middle concentration anthocyanin group: 0.3 mg/ml blueberry anthocyanin treated model cells. **e** High concentration anthocyanin group: 0.9 mg/ml blueberry anthocyanin treated model cells. Scale bar = 50 μm. **B** Mass spectrograms of five blueberry monomers.

**Additional file 3: Fig. S2** TEC reduced the expression of inflammatory factors and TLR4 in NASH. **A**–**C** The expression of inflammatory mediators was detected by ELISA. **D** The expression level of TLR4 using qRT-PCR. The values are expressed as mean ± SD.*P < 0.05, **P < 0.01, ***P < 0.001, *NS* No significance (n = 3).

**Additional file 4: Fig. S3** The interaction diagram of tsRNAs–mRNAs-pathways. Green: tsRNA, pink: mRNA, yellow: pathway.

**Additional file 5: Fig. S4** TEC reduced the expression of inflammatory factors and TLR4 in NASH by tRF-47 in vitro. **A**, **B** The expression of inflammatory mediators was detected by ELISA. **C** The expression level of TLR4 using qRT-PCR. The values are expressed as mean ± SD.*P < 0.05, **P < 0.01 (n = 3).

## Data Availability

The original contributions proposed in the study are stored in articles and Supplementary Materials, and further inquiries can be made directly to the corresponding author.

## Abbreviations

NASH: Nonalcoholic steatohepatitis
UPLC-MS: Ultra performance liquid chromatography-mass spectrometry
H&E: Haematoxylin–eosin
TEC: Tectorigenin
C3G: Cyanidin-3-O glucoside
TLR4: Toll-like receptor-4
tRF-47: TRF-47-58ZZJQJYSWRYVMMV5BO
NLRP3: NLR family pyrin domain containing 3
tsRNA: TRNA-derived small RNA
NAFLD: Non-alcoholic fatty liver disease
3MA: 3-Methyladenine
qRT-PCR: Quantitative real-time polymerase chain reaction
GO: Gene Ontology
KEGG: Kyoto encyclopedia of genes and genomes
AST: Aspartate aminotransferase
ALT: Alanine aminotransferase
MDA: Malondialdehyde
TG: Triglyceride
TC: Total cholesterol
GSDME: Gasdermin E
HFD: High-fat diet
