# Supplementary material for: The monomer TEC of blueberry improves NASH by augmenting tRF-47-mediated autophagy/pyroptosis signaling pathway
Source: J Transl Med. 2022 Mar 14;20:128. doi: 10.1186/s12967-022-03343-5 (PMC8919551; doi:10.1186/s12967-022-03343-5)
Supplement: Supplementary file 1 — Additional file 1: Table S1 The primers and sequence. Table S2 Determination of monomer components in anthocyanins by UPLC. Table S3 The top ten up and down regulated tsRNAs. [file 12967_2022_3343_MOESM1_ESM.doc]

**Table S1** The primers and sequence

| **Genes** | **5'to3'** |
| --- | --- |
| U6-F | CGATACAGAGAAGATTAGCATGGC |
| U6-R | AACGCTTCACGAATTTGCGT |
| tRF-45-7Z8L8NRS9NS334L2H1 F | tcattggtcgtggttgtagtccgtgcgaga |
| tRF-45-7Z8L8NRS9NS334L2H1 RT | GTCGTATCCAGTGCGTGTCGTGGAGTCGGCAATTGCACTGGATACGACGGTATTC |
| tRF-47-58ZZJQJYSWRYVMMV5BO F | catatcattggtcgtggttgtagtccgt |
| tRF-47-58ZZJQJYSWRYVMMV5BO RT | GTCGTATCCAGTGCGTGTCGTGGAGTCGGCAATTGCACTGGATACGACTATTCTC |
| tRF-47-2BJ65YXENDBP1IUUK7O F | ggtccagtttttttttttttttaagg |
| tRF-47-2BJ65YXENDBP1IUUK7O RT | GTCGTATCCAGTGCGTGTCGTGGAGTCGGCAATTGCACTGGATACGACTAAGGGG |
| tRF-33-87R8WP9N1EWJDW F | ggtccagtttttttttttttttagc |
| tRF-33-87R8WP9N1EWJDW RT | GTCGTATCCAGTGCGTGTCGTGGAGTCGGCAATTGCACTGGATACGACAGCGCCG |
| tRF-45-58ZZJQJYSWRYVMMV5B F | catatcattggtcgtggttgtagtccgt |
| tRF-45-58ZZJQJYSWRYVMMV5B RT | GTCGTATCCAGTGCGTGTCGTGGAGTCGGCAATTGCACTGGATACGACTTCTCGC |
| GAPDH-F | AGAAGGCTGGGGCTCATT |
| GAPDH-R | AGAAGGCTGGGGCTCATT |
| TLR4-F | AGTTGATCTACCAAGCCTTGAGT |
| TLR4-R | GCTGGTTGTCCCAAAATCACTTT |

**Table S2** Determination of monomer components in anthocyanins by UPLC-MS

| Items | C3G | Myricetin | Myricetin 3-O-galactoside | Delphinidin | TEC |
| --- | --- | --- | --- | --- | --- |
| Methanol extraction | 27.24±0.26 mg/100g | 319.60±33.16 ug/100g | 1.08±0.16 mg/100g | 8.10±0.88 mg/100g | 1.27±0.17 mg/100g |
| Water extraction | 6.24±0.73 mg/100g | 1.03±0.13 ug/100g | - | 50.34±7.92ug/100g | 0.94±0.08 ug/100g |

**Table S3** The top ten up and down regulated tsRNAs

| AccID | log_2_FC | Pvalue | Style |
| --- | --- | --- | --- |
| tRF-26-9MVH7P59N3E | 4.11466834 | 0.01222773 | up |
| tRF-19-NZD75KJ2 | 3.81532616 | 0.02372196 | up |
| tRF-27-73H3RXPLQV3 | 3.78288317 | 0.02615516 | up |
| tRF-28-FSXMSL73VLD5 | 3.69998871 | 0.0099046 | up |
| tRF-21-19BMW3RDD | 3.64441535 | 0.01138799 | up |
| tRF-23-9JKL0653DL | 3.56495299 | 0.02000832 | up |
| tRF-19-KQBRFKJL | 3.48722498 | 0.02542676 | up |
| tRF-34-HMI8W47W1R7HE6 | 3.3672386 | 0.00577431 | up |
| tRF-23-834QD7OK0K | 3.19353573 | 0.03376973 | up |
| tRF-26-V3WD8YQ84VD | 3.13171493 | 0.04583482 | up |
| tsRNA-1030 | -1.9452163 | 0.01981145 | down |
| tRF-21-QFI2VHJY0 | -1.9456663 | 0.00365575 | down |
| tRF-24-R71KVUY92E | -1.974232 | 0.04507621 | down |
| tRF-29-KSR95R3J09FV | -2.0729695 | 0.0084724 | down |
| tRF-18-245PLQ04 | -2.1814494 | 0.00328447 | down |
| tRF-23-R95R3J09D1 | -2.5366444 | 0.0010537 | down |
| tRF-29-8SR95R3J09FV | -2.5800111 | 0.00331064 | down |
| tRF-22-7JPJ60MVP | -2.6074998 | 0.03375168 | down |
| tRF-23-NNBYBKZ3DZ | -3.7236355 | 0.01958915 | down |
| tRF-24-7SDR2SN1E2 | -3.9852552 | 0.00867333 | down |
