# Supplementary figures and images for: The monomer TEC of blueberry improves NASH by augmenting tRF-47-mediated autophagy/pyroptosis signaling pathway
Source: J Transl Med. 2022 Mar 14;20:128. doi: 10.1186/s12967-022-03343-5 (PMC8919551; doi:10.1186/s12967-022-03343-5)

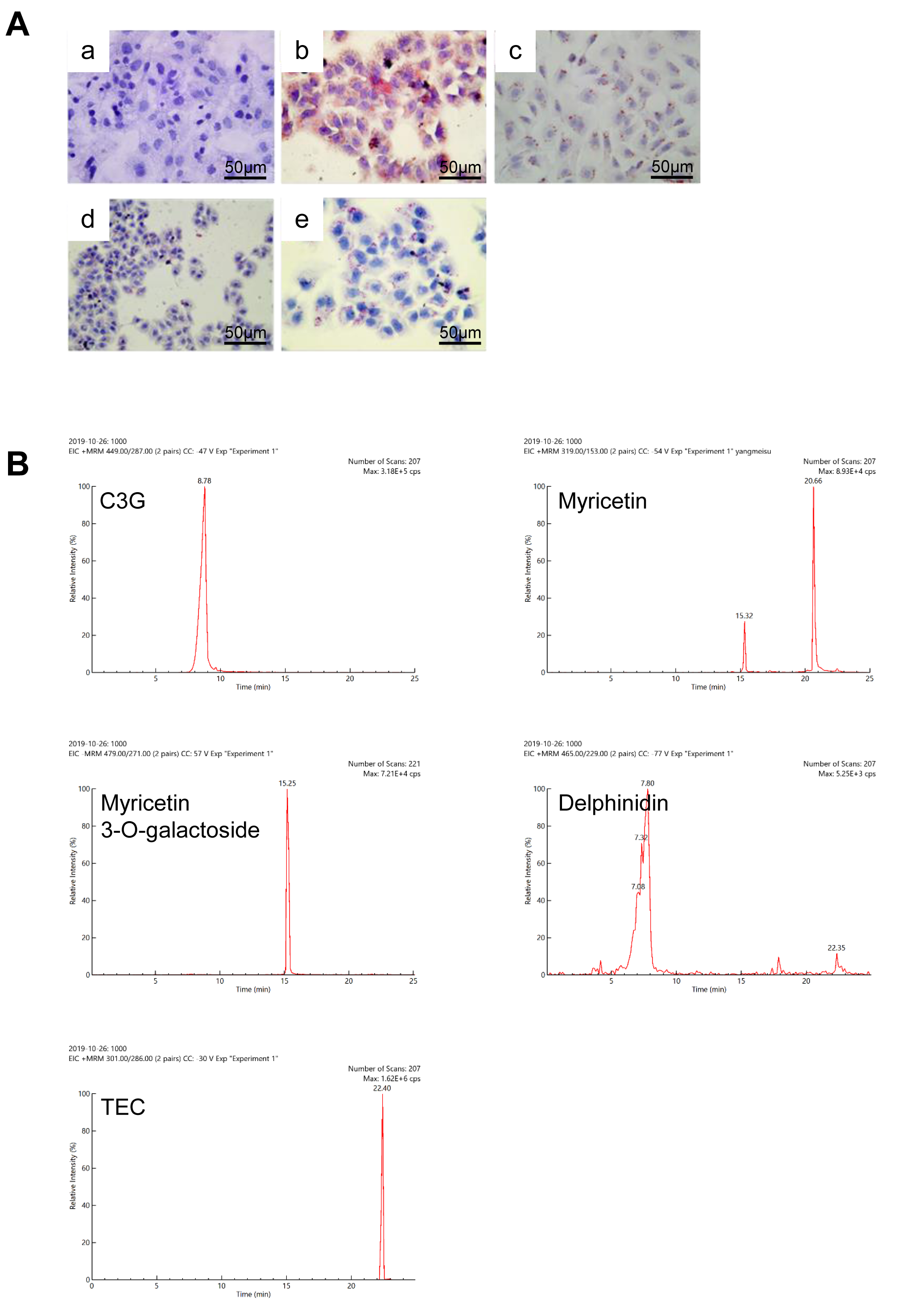

Supplement: Supplementary file 2 — Additional file 2: Fig. S1 Analysis of active components in blueberry. A The effects of different concentrations of anthocyanins on lipid droplet formation in HepG2 cells were detected by Oil Red O staining. a Blank group: cells were not treated. b NASH model group: steatosis HepG2 cells with FFA (0.5 mM) induced. c Low concentration anthocyanin group: 0.1 mg/ml blueberry anthocyanin treated model cells. d Middle concentration anthocyanin group: 0.3 mg/ml blueberry anthocyanin treated model cells. e High concentration anthocyanin group: 0.9 mg/ml blueberry anthocyanin treated model cells. Scale bar = 50 μm. B Mass spectrograms of five blueberry monomers. [file 12967_2022_3343_MOESM2_ESM.tif]

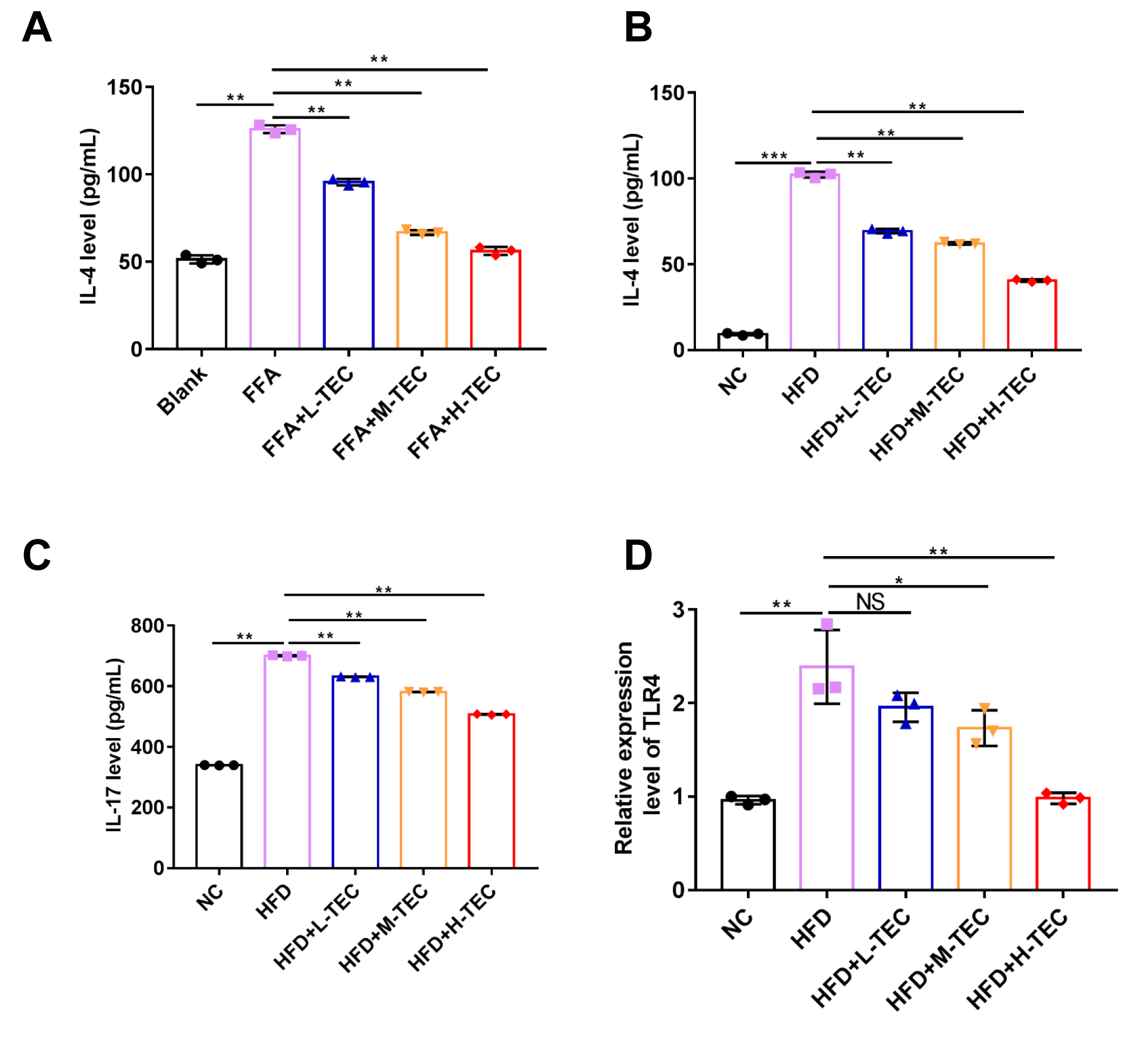

Supplement: Supplementary file 3 — Additional file 3: Fig. S2 TEC reduced the expression of inflammatory factors and TLR4 in NASH. A–C The expression of inflammatory mediators was detected by ELISA. D The expression level of TLR4 using qRT-PCR. The values are expressed as mean ± SD.*P < 0.05, **P < 0.01, ***P < 0.001, NS No significance (n = 3). [file 12967_2022_3343_MOESM3_ESM.tif]

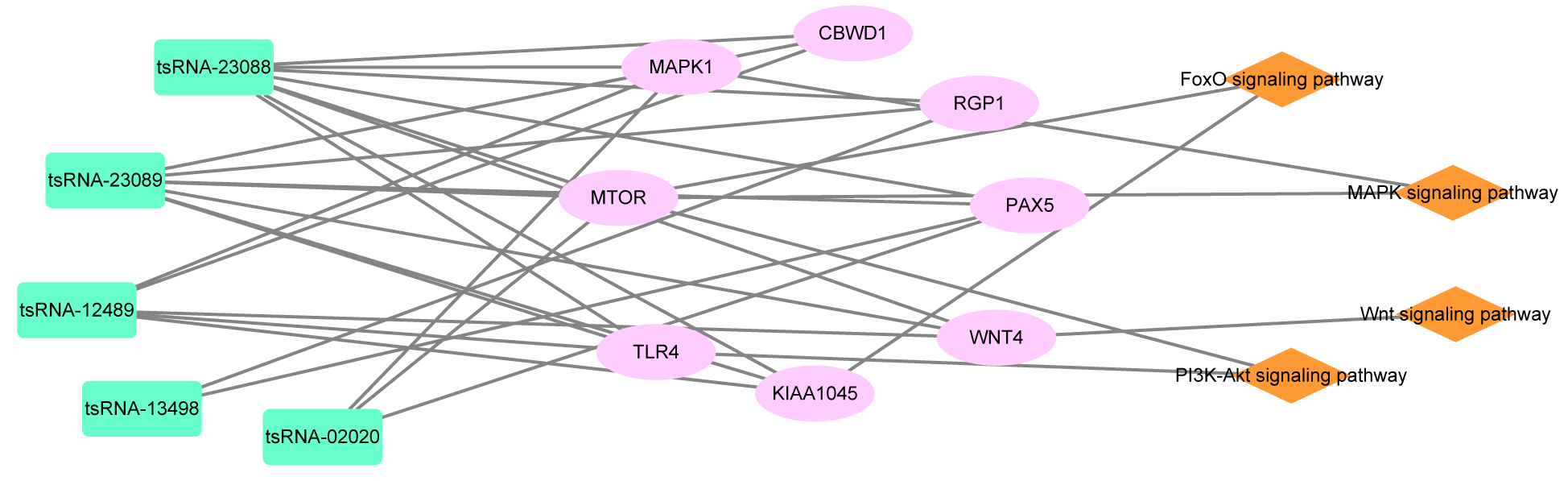

Supplement: Supplementary file 4 — Additional file 4: Fig. S3 The interaction diagram of tsRNAs–mRNAs-pathways. Green: tsRNA, pink: mRNA, yellow: pathway. [file 12967_2022_3343_MOESM4_ESM.tif]

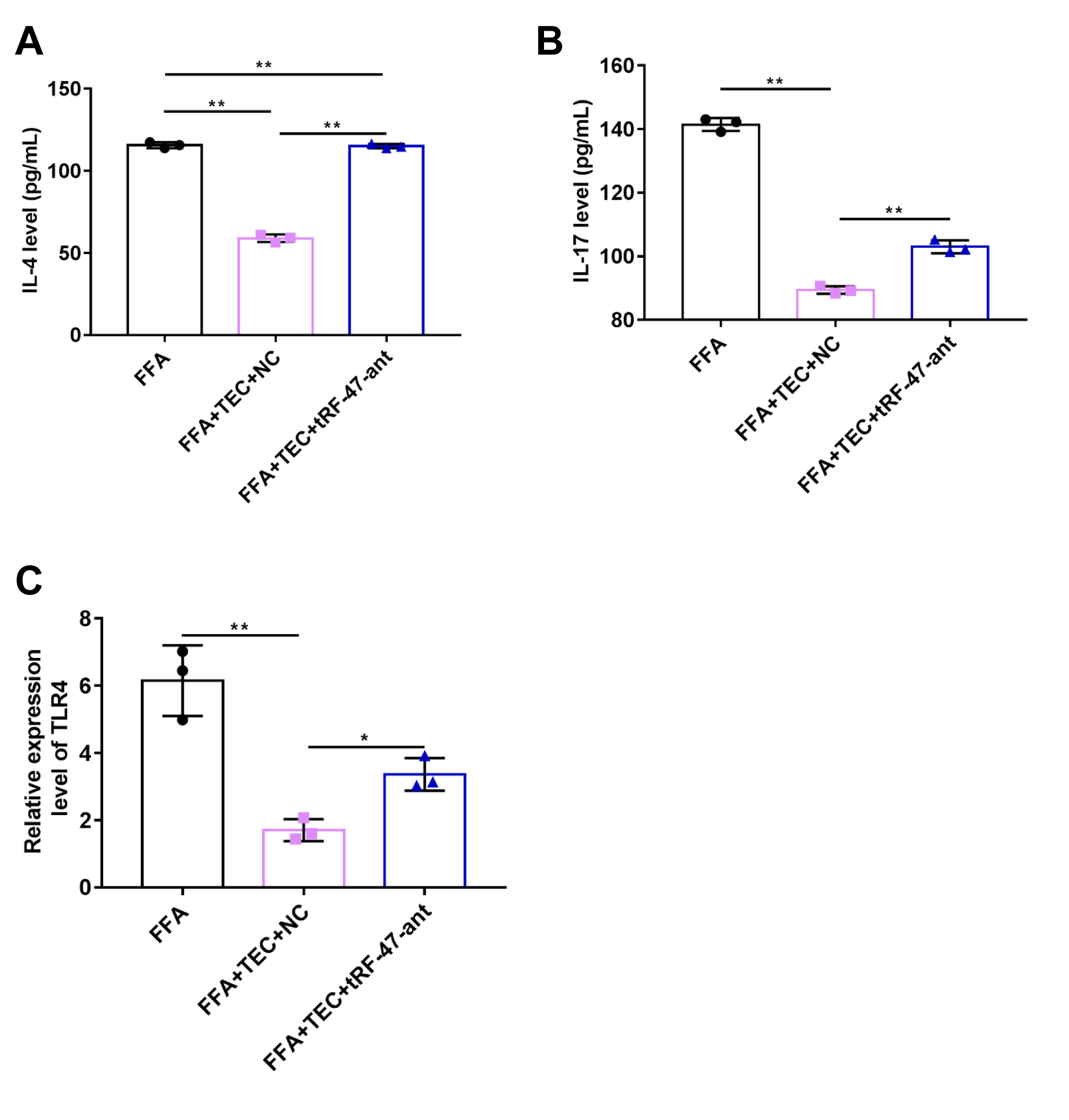

Supplement: Supplementary file 5 — Additional file 5: Fig. S4 TEC reduced the expression of inflammatory factors and TLR4 in NASH by tRF-47 in vitro. A, B The expression of inflammatory mediators was detected by ELISA. C The expression level of TLR4 using qRT-PCR. The values are expressed as mean ± SD.*P < 0.05, **P < 0.01 (n = 3). [file 12967_2022_3343_MOESM5_ESM.tif]
